# Supplementary material for: Resveratrol Alleviates Diabetic Periodontitis-Induced Alveolar Osteocyte Ferroptosis Possibly via Regulation of SLC7A11/GPX4
Source: Nutrients. 2023 Apr 28;15(9):2115. doi: 10.3390/nu15092115 (PMC10181281; doi:10.3390/nu15092115)
Supplement: Supplementary file 1 [file nutrients-15-02115-s001.zip › nutrients-2321272-supplementary.pdf]

Supplementary materials

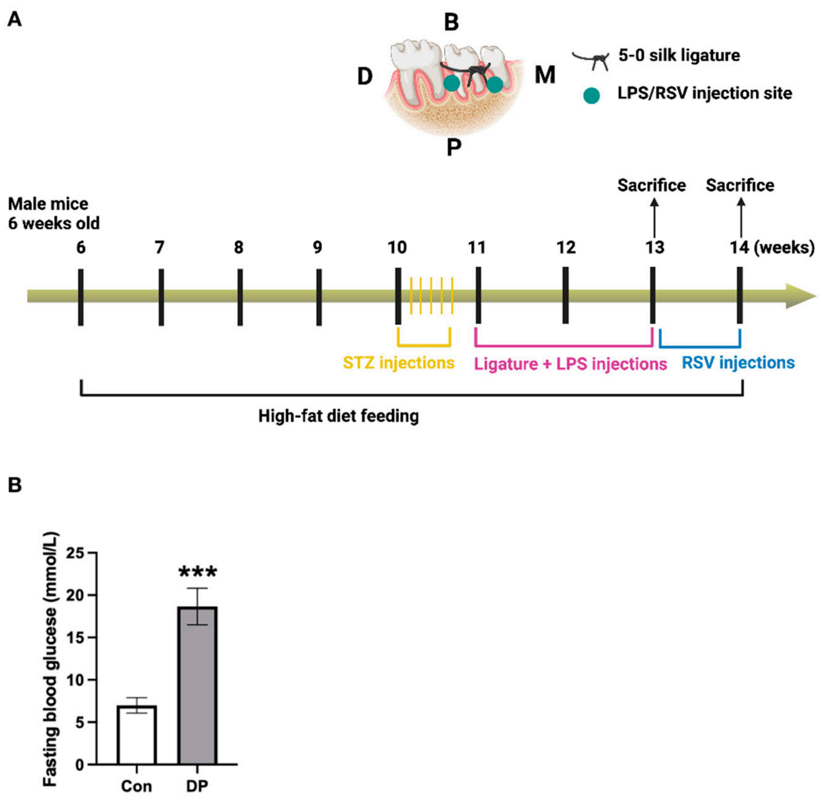

Supplementary Figure S1: (A) Flow chart of modeling of mice diabetes periodontitis disease model. (B) Blood glucose detection of mice at 12 weeks. *** $p < 0.001$.

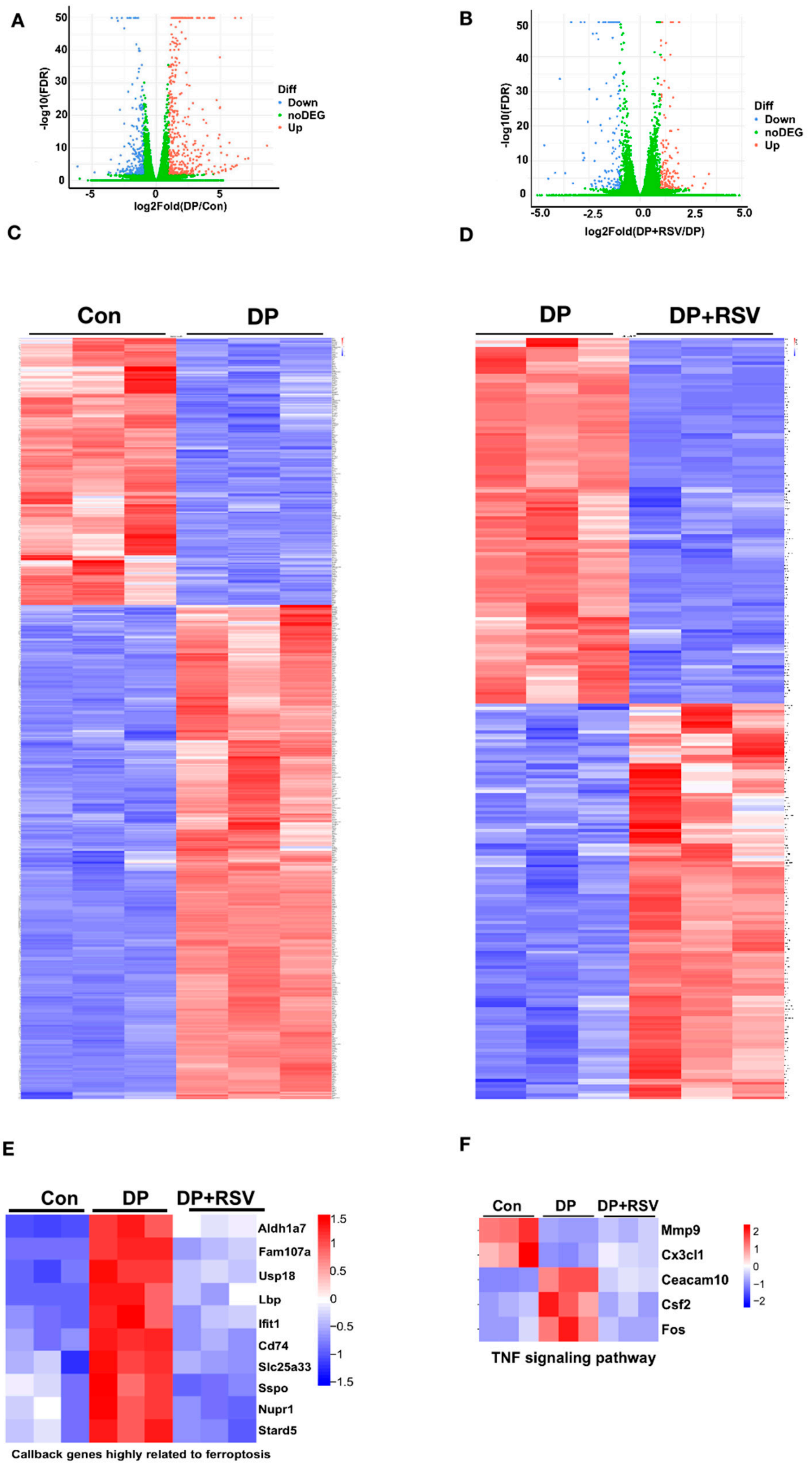
A
-log10(FDR)
log2Fold(DP/Con)
Diff
Down
noDEG
Up
B
-log10(FDR)
log2Fold(DP+RSV/DP)
Diff
Down
noDEG
Up
C
Con
DP
D
DP
DP+RSV
E
Con
DP
DP+RSV
Aldh1a7
Fam107a
Usp18
Lbp
Ifit1
Cd74
Slc25a33
Sspo
Nupr1
Stard5
Callback genes highly related to ferroptosis
F
Con
DP
DP+RSV
Mmp9
Cx3cl1
Ceacam10
Csf2
Fos
TNF signaling pathway

Supplementary Figure S2: (A) Volcano map showing differentially expressed mRNAs. in Con vs. DP. (B) Volcano map showing differentially expressed mRNAs. in DP vs. DP+RSV. (C) Heatmap of differentially expressed mRNAs. in Con vs. DP. (D) Heatmap of differentially expressed mRNAs. in DP vs. DP+RSV. (E) Heatmap of callback genes highly related to ferroptosis. (F) Heatmap of TNF signaling pathway in callback genes.

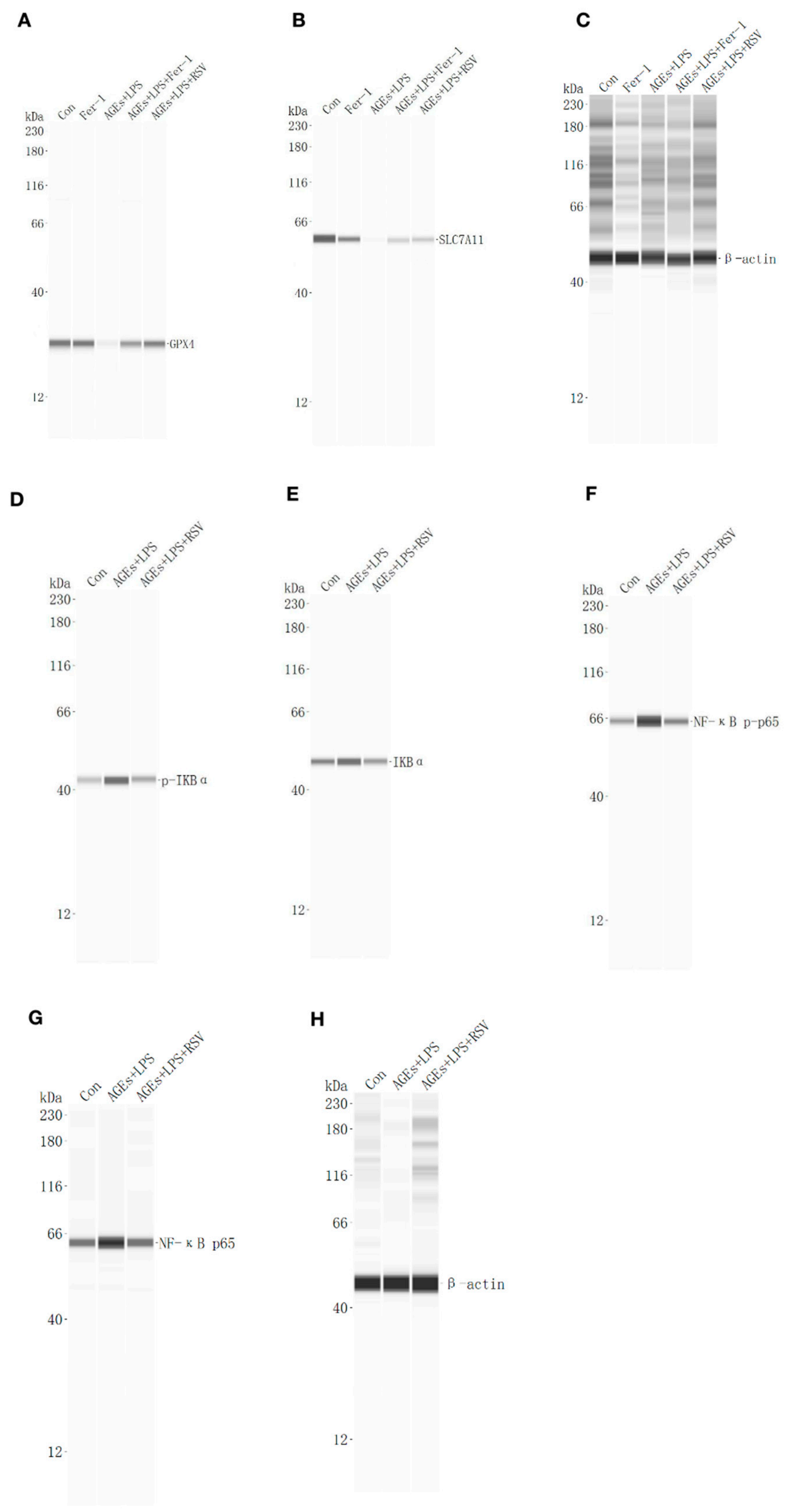
A
Con
Fer-1
AGEs+LPS
AGEs+LPS+Fer-1
AGEs+LPS+RSV
kDa
230
180
116
66
40
12
GPX4
B
Con
Fer-1
AGEs+LPS
AGEs+LPS+Fer-1
AGEs+LPS+RSV
kDa
230
180
116
66
40
12
SLC7A11
C
Con
Fer-1
AGEs+LPS
AGEs+LPS+Fer-1
AGEs+LPS+RSV
kDa
230
180
116
66
40
12
β-actin
D
Con
AGEs+LPS
AGEs+LPS+RSV
kDa
230
180
116
66
40
12
p-IKBα
E
Con
AGEs+LPS
AGEs+LPS+RSV
kDa
230
180
116
66
40
12
IKBα
F
Con
AGEs+LPS
AGEs+LPS+RSV
kDa
230
180
116
66
40
12
NF-κB p-p65
G
Con
AGEs+LPS
AGEs+LPS+RSV
kDa
230
180
116
66
40
12
NF-κB p65
H
Con
AGEs+LPS
AGEs+LPS+RSV
kDa
230
180
116
66
40
12
β-actin

Supplementary Figure S3: Original blot for Fig. 4E. Capillary-based immunoassay analysis of (A) GPX4, (B) SLC7A11, and (C) β-actin in osteocytes. Original blot for Fig. 6C. Capillary-based immunoassay analysis of (D) p-IKB*α*, (E) IKB*α*, (F) NF-κB p-p65, (G) NF-κB p65, and (H) β-actin in osteocytes.
